# Family-Based, Culturally Responsive Intervention for Chinese Americans With Diabetes: Lessons Learned From a Literature Review to Inform Study Design and Implementation

**DOI:** 10.2196/48746

**Published:** 2023-11-20

**Authors:** Wen-Wen Li, Jacqueline Tong

**Affiliations:** 1 San Francisco State University San Francisco, CA United States

**Keywords:** family-based intervention, diabetes, HbA1c, Chinese, implementation, prevalence, systematic review, patient, intervention, diabetes care, effectiveness, diabetes management, cultural intervention, patient education, Chinese Americans

## Abstract

**Background:**

The prevalence of diabetes in the United States is very high, and Chinese peoples with diabetes are estimated to comprise 50% of the total cases. Rates of diabetes continue to rise among Chinese and Chinese American people; however, research regarding effective diabetes interventions for this minority group is sparse.

**Objective:**

A literature review was conducted to determine a study design and interventions for future studies investigating the efficacy of a family-based intervention to improve diabetes care for Chinese Americans.

**Methods:**

The review was conducted from January 2023 to April 2023. The PubMed, CINAHL, ScienceDirect, ProQuest, Google Scholar, Scopus, and Cochrane Central Register of Controlled Trials databases were searched. The key search terms were “diabetes type 2,” “Chinese patients,” “minority patients,” “interventions for diabetes,” “diabetes and family,” “culturally responsive interventions for diabetic patients,” “family education for diabetes,” and “diabetes in China.”

**Results:**

The initial search retrieved 2335 articles, and 10 articles met the selection criteria to examine the efficacy of family-based interventions for Chinese American people. The review showed that providing multiple sessions of education and counseling for both patients and family members is promising for improving diabetes care. Recruitment of 20 to 60 dyads consisting of a patient and a family member can help assess family dynamics in the process of diabetes care, such as food shopping and preparation, and of diabetes management to further evaluate the efficacy of an intervention. Glycated hemoglobin (HbA_1c_) was the most often used primary outcome. Other secondary outcomes included knowledge and efficacy in diabetes management and self-care activities related to diabetes care.

**Conclusions:**

A family-based intervention is essential for optimizing diabetes care for Chinese Americans. Thus, recruitment of a dyad consisting of a patient and a family member is important to investigate the efficacy of a family-based intervention for improving diabetes care in this population. Strategies for improving recruitment and retention of dyads were identified. In addition, technology can be used to promote the delivery of interventions to patients, which in turn increases efficacy. This review can help researchers investigate the efficacy of family-based interventions for promoting diabetes management by designing culturally appropriate study protocols and interventions.

## Introduction

### Overview

Despite advances in health care knowledge and science, diabetes and its complications remain a significant issue in the United States, causing premature deaths and a huge financial burden on the society [1]. It is estimated that the prevalence of diabetes will increase to more than 54.9 million Americans in 2030; this is an increase of 54% from 2015 to 2030 [1]. In addition, the total annual medical costs related to diabetes will be more than $622 billion by 2030 [1]. Thus, diabetes management and self-management are essential areas for research. When examining the demographics of people with diabetes in the United States, it is clear that type 2 diabetes is a significant issue for minority groups such as Chinese and Chinese American adults [1]. The care of adult patients with type 2 diabetes is a complex process, particularly for minority populations in the United States.

### Problem Statement

The population of Chinese immigrants in the United States is rapidly increasing and has grown by over 2 million from 2000 to 2015 [2]. As stated, the prevalence of diabetes in the United States is projected to be 54.9 million cases in 2030, and Chinese patients with diabetes are estimated to comprise 50% of the total cases [1,3]. Rates of diabetes continue to rise among Chinese and Chinese American people; however, research regarding effective interventions for this minority group is sparse [3-5]. Current yet limited research demonstrates the potential efficacy of family support and involvement for improving diabetes management for patients among certain cultures, such as for Chinese people [3,4]. However, the findings are not robust enough to make significant clinical implications, as this area of research is rarely implemented with Chinese Americans [3,4,6]. Chinese patients usually place high value on family; thus, family structure and support can significantly impact their disease management [3]. Therefore, involving family in disease management is culturally responsive with potential effectiveness for optimizing diabetes management for Chinese Americans. Family members are defined as relatives living with the patients or in regular contact with patients at least once a week [7].

In the general US population, family support interventions showed more robust efficacy for diabetes management when compared with traditional approaches such as individual patient education on diet control and medication compliance [3]. Family-based interventions cover a broad range of activities, such as preparation of a culturally and medically appropriate diet and collective decision-making on crucial issues such as limb amputation. However, limited literature could be found on the efficacy of family support for diabetes management for Chinese Americans.

Regarding study populations, our search was expanded to include research conducted with Chinese Americans as well as other ethnic minorities, such as Hispanic and Korean Americans, who also have similar cultural values of viewing family as their important support system.

The goal of this literature review was to provide a comprehensive review of the existing literature to inform future researchers about details and specifics for study design, components of an effective family-based intervention, outcome measures, and how to efficiently recruit and retain participants within dyads of patients and family members.

## Methods

### Literature Review

The literature review was conducted between January 2023 and April 2023, and we searched the PubMed, CINAHL, ScienceDirect, ProQuest, Google Scholar, Scopus, and Cochrane Central Register of Controlled Trials databases. Keywords were “diabetes mellitus type 2,” “type 2 diabetes mellitus,” “Chinese patients,” “Chinese Americans,” “Chinese immigrants,” “minority patients,” “interventions for diabetes,” “diabetes and family,” “culturally responsive interventions for diabetic patients,” “family education for diabetes,” and “diabetes in China.” The literature search was performed by 2 reviewers using various keywords from the aforementioned list. For instance, we used a combination of “type 2 diabetes mellitus” and “Chinese Americans” and “family education” as the initial approach to search articles in PubMed. The first reviewer used various combinations of keywords for the search and documented the entire search history. The second reviewer used the same combinations of various keywords from the first reviewer and documented the search results. The number of articles for each search was compared by the 2 researchers. If there was discrepancy in terms of the numbers of articles, the 2 reviewers performed the search again and discussed the results to reach consensus (the same number of articles). This process was conducted with all databases, and the number of articles were summed to establish the first pool of papers (for the exact number, review the “Study Quality” section).

The inclusion criteria were developed by our research team members, including 2 clinicians who had been working with Chinese Americans for more than 20 years and 2 researchers who had 10 years to 15 years of research experience with Chinese Americans. The inclusion criteria included report of glycated hemoglobin (HbA_1c_) levels (a gold standard for diabetes management); recruitment of patients older than 18 years of age; patients who identified as Chinese, Chinese American, or other minority ethnicity such as Hispanic or Korean who share similar family support systems; use of a continuous family-based, culturally responsive intervention as a primary approach; or investigation of how family support helped or was associated with diabetes management. Studies were retrieved from peer-reviewed journals published in various countries and written in English. Study types ranged from quasiexperimental and qualitative to randomized control trials (RCTs).

### Study Quality

**Figure 1:**
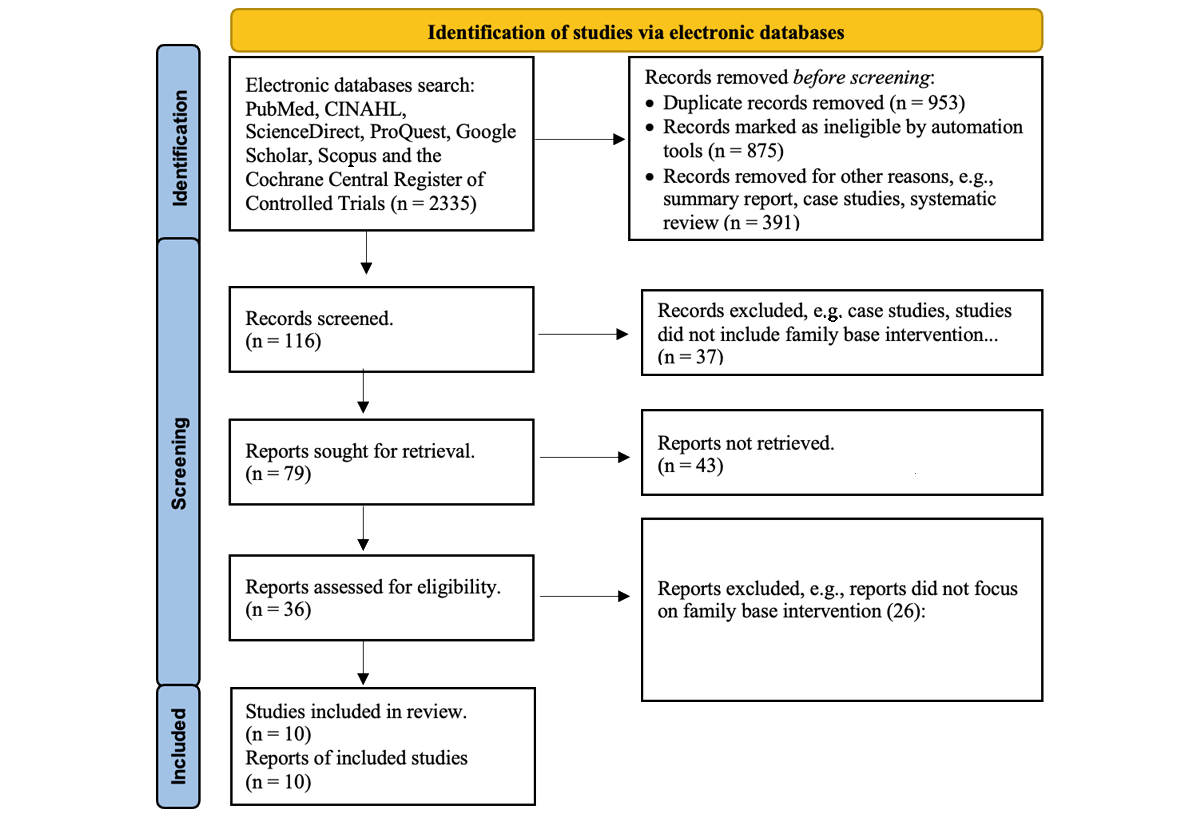
PRISMA flow chart for selected studies on family-based diabetes management.

Assessment of study quality was developed by the research team. The articles had to meet the following requirements (the standard for good quality studies) to be eligible for our final review: (1) meeting all inclusion criteria; (2) providing the full text; (3) not a summary report, case study, nor systematic review; (4) focus on family-based interventions or providing important information related to family-based interventions. Figure 1 shows the flow chart describing how the articles were screened and selected. The screening algorithm was developed by the research team by modifying the PRISMA (Preferred Reporting Items for Systematic Reviews and Meta-Analyses) flow chart [8]. The entire screening process was conducted by 2 reviewers together.

The initial search retrieved 2335 articles, and 10 articles met the selection criteria and were included in this review. These studies were conducted in various countries, such as the United States and China, and were written in English. In terms of the review process, 2 reviewers conducted their literature review independently, then met to discuss their review results and the review tables they generated. The consensus of the review results from the 2 reviewers is described in the “Results” section.

## Results

### Study Design

Regarding study design, there were 2 RCTs, 2 quasiexperimental studies, 3 cross-sectional studies, and 3 qualitative studies.

### Study Duration

Studies were conducted for a duration of time ranging from 2 months to 19 months, depending on the resources and availability of study participants. Although significant results were identified for shorter studies of around 6 months, studies lasting longer than 12 months were more reliable due to the chronic nature of diabetes and that effective management requires repeated tests throughout the patient’s lifetime.

### Study Bias

Loss of follow-up may lead to study bias as it limits generalizability of the study findings to Chinese or Chinese American patients with type 2 diabetes at large [6]. For instance, 1 study cited a loss of follow-up [6]; up to 20% of participants were unable to complete the study. The participants who withdrew from the study might have had more serious health conditions that prevented them from continuing to participate in the study. This is particularly challenging for the studies that involved both a participant and a family member who had to commit to several months of educational or interview sessions with little incentive to complete the required number of sessions. The possible solutions to promote recruitment and retention of a dyad will be discussed under in the “Incentives” section.

Another potential bias may be associated with recruitment of only specific patient populations. For example, some studies focused exclusively on elderly patients who were hospitalized [4,6], while others involved participants who had a low income or were from rural areas in China [9,10]. Thus, the generalizability of the study findings was limited. For future studies, a broader population base, such as from young to older adults and across different settings, should be included to generate more generalizable findings. This is especially true when a large sample size is available and appropriate (eg, in a more advanced stage of investigating the effectiveness of a well-established intervention instead of a newly developed, untested intervention).

### Sample Size

Sample size was reported based on the study design. For the intervention studies, the sample size ranged from 29 dyads to 225 dyads.

#### Two RCTs

The study by Feng et al [6] used a rigorous RCT design with 2 groups (225 dyads; intervention vs control): 113 patients in the intervention group and 112 patients in the control group. McEwen et al [9] also used an RCT design with 2 groups (157 dyads; intervention vs control): 83 dyads in the intervention group and 74 dyads in the control group. Feng and colleagues [6] have not published their data. McEwen et al [9] reported significant improvement of diabetes management outcomes, such as diabetes management activities, but not of HbA_1c_.

#### Two Quasiexperimental Studies

Cai and Hu [11] conducted a 2-group quasiexperimental study (without randomization of participants): 29 dyads in the intervention group and 28 dyads in the control group. Hu et al [12] conducted a 2-group quasiexperimental study (92 dyads): 51 dyads in the intervention group and 41 dyads in the control group. Both studies showed a significant reduction in HbA_1c_.

#### Three Cross-Sectional Studies

Recruitment in the cross-sectional studies resulted in the following samples: 70 dyads in the study by Zhao et al [7]; 532 participants (patients with type 2 diabetes) in the study by Shao et al [10]; 83 participants (baseline survey data from an original RCT) in the study by Song et al [13]. All 3 studies reported that family support was positively associated with diabetes control (eg, a better HbA_1c_ outcome).

#### Three Qualitative Studies

Recruitment in the qualitative studies resulted in the following samples: 229 participants in the study by Chen et al [4]; 20 dyads in the study by Chesla et al [3]; 20 participants in the study by Yue et al [5]. All 3 qualitative studies described in detail how and why family support was associated with diabetes management.

### Inclusion Criteria and Recruitment

The inclusion criteria commonly used across most studies involved a current diagnosis of type 2 diabetes, age older than 18 years, recent HbA_1c_ value ≥7%, and presence of an adult family member willing to participate in the study with the patient. Shao et al [10] also stipulated that patients had no concurrent malignant tumor, type 1 diabetes, gestational diabetes, nor acute complications of type 2 diabetes to avoid incidence of further complications that may have biased the study findings. For future studies, these inclusion criteria were recommended to obtain more unbiased and reliable outcomes for diabetes management.

In terms of sources for recruiting participants, the review showed that the majority of the studies recruited their participants through the community [3,4,6,7,9,11-13] and referrals from health care providers [4,5,11,12]. The literature also showed that the best strategies to recruit and retain Chinese Americans are through outreach to communities and via referral by health care providers [14].

### Incentives

As discussed earlier, incentives improved health care outcomes for patients with type 2 diabetes and reduced the number of participants lost to follow-up, especially for older Chinese adults without an income or who were living with a compromised financial situation [4,15]. Cai and Hu [11] provided incentives of free physical examinations and finger stick blood glucose tests at the end of their intervention period, which led to a low attrition rate. In addition, they instructed participants to communicate within their assigned smaller groups to remind each other about upcoming education sessions and follow-up events. Offering incentives and peer support for participation in the study also significantly reduced the attrition rate. For example, Cai and Hu [11] had an attrition rate of 5% using their peer support.

### Intervention

Feng et al [6] aimed to examine the effects of a family-based intervention utilizing the Chinese social media application WeChat. WeChat is a messaging system that includes news and other information that is periodically distributed to participants. This was a 2-armed RCT following 225 dyads of Chinese patients and their family members. Their intervention provided educational articles via WeChat over a period of 12 months. The control group continued to receive regular medical care. The educational articles were divided into 2 themes: (1) optimal diets for patients with diabetes and (2) exercise recommendations [6]. Compared with those in the control group, the patients in the intervention group achieved significant changes in HbA_1c_, self-care activities, and risk perception of diabetes.

McEwen et al [9] also implemented a 2-armed RCT to examine the efficacy of a 12-week educational intervention for Hispanic patients and their families. The research team conducted a group session intervention for the participant-family member dyads that focused on materials from the National Standards for Diabetes Self-Management Education [12]. Group sessions were held to discuss tips and challenges with diabetes management and how to overcome challenges. McEwen et al [9] implemented an extensive intervention protocol, including 3 weekly home visits, 3 weekly follow-up phone calls from a health care practitioner, and 6 group sessions over 12 weeks for the intervention group. The comparison consisted of wait list control groups. Compared with other studies, McEwen et al [9] used a greater variety of interventions, including patient education, family support, and telephone calls to follow up on home visits. However, their intervention was very labor-intensive. Compared with the control group, the intervention group achieved better diabetes management, except HbA_1c_ levels (Table 1).

**Table 1:** Description of studies included in the systematic review.

Author(s), year	Study purpose	Study design and sample size	Intervention	Participants and inclusion criteria	Recruitment	Outcomes
Zhao et al, 2022 [7]	To examine the effects of the patients’ and family members’ perceived family support on diabetes management	Cross-sectional; 70 dyads	N/A^a^	Chinese people in China DM^b^ ≥6 months Aged 50-79 years HbA_1c_^c^ between 7.0% and 10.0%	Recruitment took place in 26 residential communities; no other details were provided.	Patients’ perceived family support, such as actively helping patients adjust their lifestyles (healthy diet and regular exercise), was positively associated with diabetes management.
Feng et al, 2023 [6]	To examine the effectiveness of a family-based intervention with WeChat	RCT^d^ with 2 groups; 225 dyads (intervention: 111; control: 111)	12-week interventions, including distribution of educational articles, quizzes, and more via WeChat (social media) to patients and family members of patients	Chinese people in China Registered in 1 of the 2 community health service centers in the urban area in Jiading District Had been diagnosed with T2DM^e^ by a doctor at least 6 months before study enrollment Aged 18 to 79 years HbA_1c_ level ≥7% Had no plans to leave their place of residence in the following 12 months Had a family member who could use WeChat and lived with the patient or visited them at least once a week	Recruited from family doctors in 2 community health centers; patients and family interviewed by the research team member	HbA_1c_ significantly decreased by 0.60% (*P*<.001); other secondary outcomes included decrease in nonsupportive behavior (*P*=.03) and improved scores for general diet (*P*<.001), specific diet (*P*<.001), exercise (*P*=.002), blood sugar testing (*P*=.02), foot care (*P*<.001), risk knowledge (*P*<.001), personal control (*P*<.001), worry (*P*=.02), optimism bias (*P*=.03), and supportive behaviors (*P*<.001).
Cai and Hu, 2016 [11]	To examine the effects of a family-based self-management education intervention for adults	Quasiexperimental design with 2 groups; 57 dyads (intervention: 29 dyads; control: 28 dyads)	7 educational sessions with patients and their family members	Chinese people in China Self-reported diagnosis of T2DM >18 years old HbA_1c_ >7.0%	Recruited via flyers posted in health community centers and via referral from physicians	Significant average 1% reduction in HbA_1c_ (intervention group vs control group); significant reduction in average BMI (23.27, SD 2.13 for the intervention group vs 24.96, SD 3.07 for the control group; *F*=18.11; *P*<.001); significant increase in average diabetes knowledge (22.97, SD 2.03 for the intervention group vs 14.11, SD 4.57 for the control group; *F*=92.77; *P*<.001).
Shao et al, 2017 [10]	To determine if better social support perceived or received by patients could reinforce self-efficacy, medical regimen, and glycemic control	Cross-sectional study; n=532	N/A	Chinese people in China Diagnosis of T2DM at least 1 year prior >18 years old No concurrent serious health conditions, such as advanced renal failure Chinese inhabitants of Guangzhou, China Able to complete a questionnaire	Recruited from outpatients and inpatient units in 2 hospitals	Social support was significantly associated with diabetes control. Social support was measured in 3 dimensions: (1) objective support (ie, visible support, such as social network), (2) subjective support (ie, an individual’s sense of being supported or understood by family), (3) support utilization (ie, the extent of accepting help and actively looking for support from family).
Chen et al, 2018 [4]	To identify common factors affecting diabetes management	Qualitative study using thematic analysis (TCA); n=229	Cross-sectional study (no intervention)	Chinese people in China Patients with T2DM >18 years old Permanent residents of Tianjin, China	Referred by physicians	Major factors (themes) related to family support for diabetes management included a higher level of family support to promote diabetes management and more social integration (eg, patients felt less lonely and more willing to optimize their diabetes control).
Hu et al, 2014 [12]	To test the efficacy of a family-based, culturally tailored intervention (8 weeks) for Hispanics with T2DM and their families	Quasiexperimental long-term study; 92 dyads (51 in the intervention group; 41 in the control group)	8-week educational sessions taught in Spanish to patients and their family members	Hispanic Americans Hispanic patients with T2DM and their family members Community-dwelling Self-identification as Hispanic Aged 18 years or older Self-identification as having a medical diagnosis of T2DM An adult family member willing to participate in the study Inclusion criteria for family members: Residence in the same household as the participant with diabetes Aged 18 years or older	Recruited from community clinics, physician offices, and churches	Mean HbA_1c_ significantly decreased by 1%, BMI significantly decreased (mean difference=–0.25 kg/m^2^; *P*=.02), and diabetes knowledge significantly improved (mean difference=5.89; *P*<.001).
McEwen et al, 2017 [9]	To investigate the effects of a family-based self-management support intervention (12 weeks) for T2DM	RCT with 2 groups; 157 dyads (83 dyads in the intervention group; 74 dyads in the control group)	Participants randomly assigned to an intervention	Mexican Americans Mexican American patient Diagnosed with T2DM at least 1 year prior 35-74 years old Spoke and read Spanish or English HbA_1c_ ≥8.0% Had not participated in a diabetes education program in the prior year Able to walk at least 1 mile (determined by self-report) Access to and ability to talk on the telephone Had 1 adult family member willing to participate	Recruited via outreach activities to various communities by the research team	HbA_1c_ did not change significantly over time, but other outcomes, including diabetes management activities and diabetes self-efficacy, were improved.
Yue et al, 2018 [5]	To explore factors affecting patients with diabetes and develop family-based interventions	Qualitative descriptive approach; 20 participants	Qualitative data collection through interviews (no intervention)	Chinese people in China HbA_1c_ target <7% T2DM diagnosis >18 years old Living in the target community	Referred by a community health care nurse to the research team	Six major themes or patient concerns were identified through data analysis that included family experiences about starvation (starvation experience in early life led to overeating, which was associated with poor diabetes management), seeking family harmony in eating, and family financial burden.
Chesla et al, 2009 [3]	To describe cultural and family challenges with illness management	Qualitative, interpretive, comparative study; 20 dyads	Qualitative data collection through interviews (no intervention)	Chinese Americans T2DM for at least 1 year Aged 35-75 years Married for a minimum of 1 year Self-identified as Chinese American or Chinese having immigrated to the United States from mainland China or Hong Kong Spouse agreed to participate	Recruitment from community clinics, community service organizations, and public notices	The results showed that Chinese families were involved in diabetes management activities, such as food preparation.
Song et al, 2012 [13]	Determine primary sources of social support for Korean Americans with T2DM and examine the effect of unmet needs in diabetes management	Baseline data from an RCT; 83 patients with DM	Survey/cross-sectional data from an RCT (no intervention)	Korean Americans Self-identified as Korean American Aged ≥30 years Uncontrolled diabetes (HbA_1c_ ≥7.5% within the past 6 months) Resided in the Baltimore, WA area Able to give written consent to participate in the study	Recruitment via social media, Korean churches, and grocery stores	The primary source of social support was their spouse (family support) for both genders; social support (mainly family support) was significantly associated with diabetes management. Diabetes management was measured by self-care activities, including diet, exercise, blood glucose testing, foot care, and medication adherence.

^a^N/A: not applicable.

^b^DM: diabetes mellitus.

^c^HbA_1c_: glycated hemoglobin.

^d^RCT: randomized controlled trial.

^e^T2DM: type 2 diabetes mellitus.

Cai and Hu [11] recruited 57 dyads and utilized a quasiexperimental 2-group design with no randomization of participants. Participants were placed in the intervention or control group based on geographic location. The intervention group required both participants and family members to undergo 7 educational sessions, including 2 one-on-one home visits and 5 group sessions. Each session focused on a particular theme, such as general knowledge, diet, physical activities, medication management, blood glucose checks, and complication prevention [11]. The control group received usual care, which included a 10-minute to 15-minute quarterly home visit for family health promotion. At the end of the study (3-month follow-up), patients in the intervention group achieved significantly better results than the control group in improving HbA_1c_, BMI, and diabetes self-care knowledge (Table 1).

Hu et al [12] recruited 92 dyads and utilized a quasiexperimental 2-group design with no randomization of participants. The intervention group participated in 8 weekly interactive modules on diabetes management including the topics of general knowledge about diabetes, facilitation of family support, identification of barriers to diabetes self-management, discussion of how to adjust lifestyle and medication to enhance diabetes control, and goal setting for healthy behaviors. The control group received 8 weeks of regular care, such as a physical exam and education about general health (eg, weight control and smoking cessation). At the end of the study (6-month follow-up), patients in the intervention group achieved significantly better results than the control group in improving HbA_1c_, lowering BMI, and increasing diabetes knowledge (Table 1).

Although the 3 cross-sectional studies, including those by Zhao et al [7], Shao et al [10], and Song et al [13], did not include any family-based interventions, their study findings highlighted important factors that were associated with diabetes management and should be considered for future family-based interventions. Zhao et al [7] reported that perceived family support by the patient, such as actively helping them adjust their lifestyles (healthy diet and regular exercise), was positively associated with diabetes management. Song et al [13] reported that family support was positively related to self-efficacy in diabetes management, including diet, exercise, and general self-management skills. They defined family support as the support that patients wanted and received from family: the more family support patients received, the better self-efficacy the patients had. Shao et al [10] also had similar findings. They further quantified family support in 3 dimensions: (1) objective support (ie, visible direct assistance, such as food preparation); (2) subjective support (ie, an individual’s sense of being supported or understood by family); (3) support utilization (ie, the extent of accepting help and actively looking for support from family). Above all, having family work with the patient to support them the way they needed and being perceived or accepted by the patient is important to develop an effective intervention in diabetes management. This also further validates our hypothesis that recruitment of dyads of a patient and a family member is crucial.

Similarly, the 3 qualitative studies by Chen et al [4], Chesla et al [3], and Yue et al [5] did not include any family-based interventions, but their study findings validated the importance of family assistance in supporting diabetes management. The examples of family support included food prepared by family, family harmony in eating, and social integration when making decisions, such as planning a diabetes management regimen together.

### Outcomes and Measurements

The most used gold standard outcome was HbA_1c_ [6,9-12]. Other outcomes related to diabetes management, such as BMI, waist circumference, diabetes knowledge, diabetes management activities, self-efficacy in diabetes management [6,9-12], and qualitative outcomes addressing how and why family factors supported diabetes management [3,4], were also included. The following studies described the efficacy of a family-based intervention for improving diabetes outcomes.

The RCT by Feng et al [6] provided educational articles via WeChat over a period of 12 months and showed a 0.6% improvement in HAb_1c_. Other outcomes were also improved, including decreased nonsupportive behavior and improvement in diet, exercise, blood glucose testing, foot care, risk knowledge, personal control, and supportive behaviors [6].

The 12-week RCT by McEwen et al [9] used a variety of interventions and did not show improvement in HbA_1c_ for Hispanic participants [9]. However, other outcomes, such as diabetes management activities and diabetes self-efficacy, were significantly improved.

The quasiexperimental study by Cai and Hu [11] showed that a family-based intervention (7 weeks) reduced patient HbA_1c_ levels by an average of 1%. This 1% reduction in HbA_1c_ has clinically significant benefits, which will prevent severe complications, such as renal failure [11]. Other outcomes, including reduced BMI, waist circumference, and improvements in diabetes knowledge, were also noted.

The quasiexperimental study by Hu et al [12] also showed significant effects of their family support intervention (ie, HbA_1c_ decreased 1%) for Hispanic participants. Other outcomes, such as BMI and diabetes knowledge, were also improved.

In terms of data driven from cross-sectional studies [7,10,13] and qualitative projects [3-5], as aforementioned, family support was highly valued by patients; thus, a healthy family dynamic should be incorporated into diabetes management to promote efficacy. For example, seeking family harmony in eating by shopping for healthy food and preparing food and eating meals together (eg, Chinese culture focuses on communal eating) can significantly optimize diabetes control. Other benefits are that patients feel less socially isolated and achieve greater efficacy at managing their diabetes.

## Discussion

This literature review attempted to provide analysis and observation of appropriate study designs to investigate the efficacy of a family-based intervention for improving diabetes management for Chinese American adults with type 2 diabetes. This discussion includes conclusions and recommendations for specific elements to design future projects, including sampling and recruitment, incentives for promoting recruitment and retention, interventions, outcomes and measurement, and study duration.

### Best Diabetes Intervention in Review

The most efficacious intervention was by Cai and Hu [11] who used a quasiexperimental 2-group design (57 dyads). The 7-week intervention resulted in a significant 1% reduction in HbA_1c_, which can lead to a 40% decreased risk of complications such as eye and kidney diseases [1]. Participants also increased their diabetes knowledge and reported improved physical and mental health. Patients felt more cared for by their family and an increased sense of community. Hu et al [12], also using a quasiexperimental 2-group design (92 dyads), had a similar intervention of an 8-week educational session provided to patients and family members in their chosen language. Patients in the intervention group had an average 0.41% decrease in HbA_1c_ at 1 month from the initial intervention. BMI and diabetes knowledge were also significantly improved. Both studies used either one-on-one or interactive modules with patients and family to facilitate family support for diabetes management. They also used various approaches, such as video games, illustrations, and educational flip charts, to facilitate understanding and implementation of the strategies that were taught for managing diabetes.

It is worth noting that the use of technology to deliver an intervention is a current trend due to its contact-free nature and good efficacy. If participants are prepared or familiar with using technology, positive outcomes are anticipated. For instance, the 12-week delivery of diabetes educational materials by Feng et al [6] achieved significant improvement of HbA_1c_ and other outcomes (eg, healthy diet). Thus, besides the strategies provided by Cai and Hu [11] and Hu et al [12], use of technology, such as WeChat (social media) as proposed by Feng et al [6], should also be considered when designing an intervention to deliver the educational information more effectively without the limit of physical distance or the impact of a pandemic. To facilitate use of technology-based interventions to further enhance optimal management of diabetes for Chinese American patients, including a younger adult family member who is savvy at using technology is especially important, as many patients are older and uncomfortable using technology to manage their disease [14,16].

Qualitative study findings also provided important information to design a family-based intervention. For instance, family support for diet and food was highly valued by patients. Thus, an intervention should contain discussion with patients and their families about food shopping and preparation provided by family, eating meals with the family in a harmonious environment, helping patients and their families find resources to resolve financial burdens in preparing healthy food or seeking medical treatment for diabetes, and facilitating healthy interactions and communication between patients and their families. Future studies should develop interventions focusing on dynamics and interactions between patients and their families instead of approaching each one separately. It is also important to understand both patient and family concerns in dealing with diabetes and help resolve those issues.

### Recruitment of Chinese Americans, Sample Size, and Incentives

In terms of sources for recruiting participants, a dyad set of a patient with diabetes and their family member was most feasible through community settings, such as a local church or community center, instead of other approaches, such as hospitals or online recruitment, due to better social connections and attachments in those settings [4,5,7,10,11]. In addition, our review demonstrated that recruitment via personal contact, such as a referral from a participant’s health care provider, will be more successful, with increased rates of participation and retention [4-6,10,11]. Other methods including public flyers or phone calls made by a research team member were not as efficient as community settings or personal referrals [4-6,10,11].

Regarding sample size, 20 to 60 dyads should be sufficient, as demonstrated by Cai and Hu [11] and Hu et al [12], in an under-studied research field, such as investigation of efficacy of family-based diabetes interventions for Chinese Americans. It is also worth noting that it is more feasible to recruit and retain a smaller number of dyads when both a patient and a family member are needed in the early stage of developing a new family-based intervention. Recruitment of dyads requires commitment. A larger sample size (eg, more than 60 dyads) is recommended for future studies with a clearer research direction (eg, a well-developed intervention) supported by robust literature to examine the effectiveness of family-based interventions that will help inform clinical practice or policy.

Incentives are important to recruit and retain study participants. Besides offering a physical examination, laboratory tests, and peer support, as mentioned in the Results section, the first author’s previous study also showed that frequent, smaller incentives, such as a gift card (in a smaller amount), throughout the study instead of one incentive at the end of the study can reduce attrition [15]. This is especially true for studies recruiting both patients and their family members as dyads [12,15].

### Outcomes

For future studies focusing on family support for Chinese Americans managing diabetes, HbA_1c_ should be used as a primary outcome accompanied by other secondary outcomes. Other measures could also include BMI, diabetes knowledge, self-efficacy in diabetes management, and self-care activities, including diet, exercise, blood glucose testing, foot care, and medication adherence.

### Conclusion

Overall, this review focused on emerging literature supporting interventions for Chinese American patients with type 2 diabetes and involving a family-based intervention.

According to the literature, the interventions involving at least one family member for diabetes care showed efficacy in improving diabetes management for Chinese Americans. However, these studies were still very limited in terms of establishing robust findings. Thus, more studies are needed in this research area.

Our review detailed information about the potential elements that should be considered when designing a family-based intervention, such as an in-person consultation with both patients and their families at the same time followed by periodic follow-up visits or phone calls. In addition, focusing on family dynamics and family support for lifestyle modifications, such as shopping for food together, is very important to promote diabetes management for Chinese Americans, as this population highly values support from family. Any family-related issues, such as no time for self-care due to spending extensive time baby-sitting grandchildren or other family members, should also be discussed for possible solutions.

Since family is highly valued by the Chinese, it is essential to recruit dyads of both a patient and an adult family member to study diabetes management instead of solely recruiting patients. Although recruiting dyads takes more time and can be challenging, strategies such as frequent incentives can promote recruitment and retention of participant dyads.

Finally, technology can be incorporated due to its advantages of saving time and no physical limitations (eg, participants do not need to commute long distances for study participation). An additional benefit of using technology is the use of a social media platform to facilitate 3-way communications among a researcher, a patient, and a family member, which can also facilitate participation by family members. However, studies using technology to help Chinese Americans manage their diabetes are very limited. Thus, this could be a future study direction for Chinese Americans with diabetes.

## Abbreviations

HbA_1c_: glycated hemoglobin
PRISMA: Preferred Reporting Items for Systematic Reviews and Meta-Analyses
RCT: randomized clinical trial
